# Sleep Disorders in Multiple Sclerosis Patients Following Diagnosis: Prevalence, Contributing Factors

**DOI:** 10.1192/j.eurpsy.2025.2344

**Published:** 2025-08-26

**Authors:** Y. Trabelsi, N. Halouani, I. Chaari, M. Turki, S. Ellouze, M. Dammak, J. Aloulou

**Affiliations:** 1 psychiatry department, hedi chaker hospital; 2 neurology department, habib Bourguiba Hospital, sfax, Tunisia

## Abstract

**Introduction:**

Sleep disorders are commonly reported among patients diagnosed with multiple sclerosis (MS), particularly in the period following the disclosure of the diagnosis. The combination of psychological stress and the chronic nature of the disease contributes to significant sleep disturbances. Understanding the prevalence, causes, and implications of these disorders is crucial for improving patient care.

**Objectives:**

The aim of this study is to evaluate the prevalence and characteristics of sleep disorders in MS patients following the announcement of the diagnosis and to explore the contributing factors, including psychological and physical symptoms.

**Methods:**

A descriptive and analytical cross-sectional study was conducted from March 1, 2023, to January 30, 2024, involving patients diagnosed with multiple sclerosis (MS)who were observed in the neurology department at Habib Bourguiba University Hospital in Sfax. We evaluated sleep disorders using the **insomnia severity index scale** which included 7 items to investigate sleep disturbance.

**Results:**

A total of 100 patients, with a mean age of 38 years ± 10 years, were included, with a strong majority of women (73%). No psychiatric history was noted. The median disease duration was 7.38 years (3-10.75). Among our patients, 64(64%) had attained a university level of education. In addition, 40(40%) were married, and 60 (60%) were single, divorced, or widowed.

The median Insomnia Severity Index was 5 (1-10.75). More than half of our patients (62%) did not suffer from insomnia. However, 9% had a mild subclinical form of insomnia, 10% had moderate clinical insomnia, and 19% suffered from severe clinical insomnia.

Primary education level was correlated significantly with mild sleep disorders n=33.3 (33.3%), (p=0.044). On the other hand, those with a higher education level showed a lower prevalence of mild sleep disorders n=22.2(22.2%) (p=0.01). However, a lower prevalence of severe sleep disorders was noted among married individuals (10.5% p=0.004), while a higher frequency was observed in divorced individuals (15.8%, p=0.). Only refusal of the diagnosis upon its initial announcement was significantly associated with severe sleep disorders (63.2%, p=0.012).

**Conclusions:**

Addressing sleep disorders in MS patients requires a holistic approach that incorporates both psychological and symptom management strategies. Early interventions targeting anxiety, depression, and physical discomfort are essential for improving sleep quality and overall well-being in this patient population. Further research is needed to develop tailored therapeutic approaches that address the unique challenges faced by MS patient’s post-diagnosis.

**Disclosure of Interest:**

None Declared

